# A Case Report of Madelung's Disease

**DOI:** 10.1055/a-2122-6121

**Published:** 2023-10-05

**Authors:** Bo Hyun Lee, Young Mann Lee, Seong Oh Park, Lan Sook Chang, Youn Hawn Kim

**Affiliations:** 1 Department of Plastic and Reconstructive Surgery, College of Medicine, Hanyang University, Seoul, Republic of Korea

**Keywords:** lipoma, lipomatosis, Madelung's disease, case reports

## Abstract

Madelung's disease (MD) is a rare disease characterized by diffuse, nonencapsulated, multiple fat masses in different areas of the body. In this case report, we present a case of MD in Asia and its management. A 66-year-old man with a history of hypertension presented with massive growth of soft tissue around the neck, breasts, upper back, and lower abdomen. Preoperative magnetic resonance imaging revealed remarkably hypertrophic fat tissue around the neck and anterior chest was wall, which consistent with the diagnosis of MD. Multiple linear incisions were made on the neck and 763, 186, 635 g of posterior, right, and left fat tissues were excised, respectively. A single wide, transverse incision was done to excise 1,072 g of fat from the upper back. Masses of both breasts were excised, preserving the inferior pedicle, weighing 1,086 (right) and 1,164 g (left). The recovery was optimal and the patient was discharged without complications. In this case, we excised the adipose masses as much as possible and improved contour and symmetry. However, the fat infiltrations in the patient were diffusely distributed, making total fat excision difficult. This rare case report may help in managing patients with MD.

## Introduction

Madelung's disease (MD) is a rare disease characterized by diffuse, nonencapsulated, multiple fat masses in the face, neck, and other areas of the body. This disorder, also known as Launois–Bensaude syndrome, benign symmetric lipomatosis, and multiple symmetric lipomatosis, was first described by Brodie in 1846 and systematically described by Madelung in 1888. 1

The incidence of MD is 1:25,000, and it mainly affects men between 30 and 60 years of age from the Mediterranean region (male-to-female ratio of 15:1), although a few cases in Asia have been reported. 2 Most patients had no family history of the disease. Chronic alcoholism was associated in 90% of cases; however, the relationship between alcohol consumption and MD remains uncertain. 3 Although the cause of MD is unknown, the involvement of catecholamines as the cause of abnormal lipogenesis has been proposed. 4

In this report, we describe a case of MD in Asia treated in our department and a review of the literature.

## Case

**Fig. 1:**
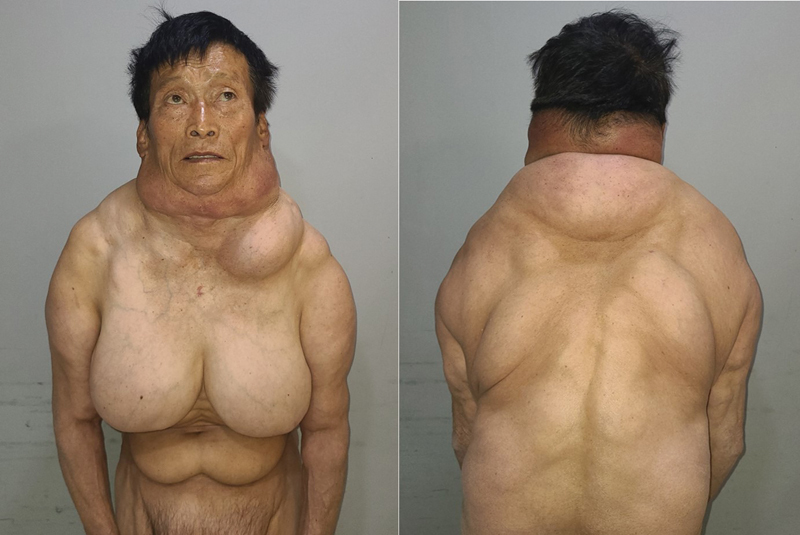
Preoperative images. A 66-year-old man with multiple systemic lipomatosis.

A 66-year-old man, known hypertensive, presented with extensive soft tissue growth around the neck, breasts, upper back, and lower abdomen ( Fig. 1 ). Growth in neck volume was noticed over several years, with no history of dyspnea or dysphagia. He was a nonsmoker and a moderate alcoholic beverage drinker.

**Fig. 2:**
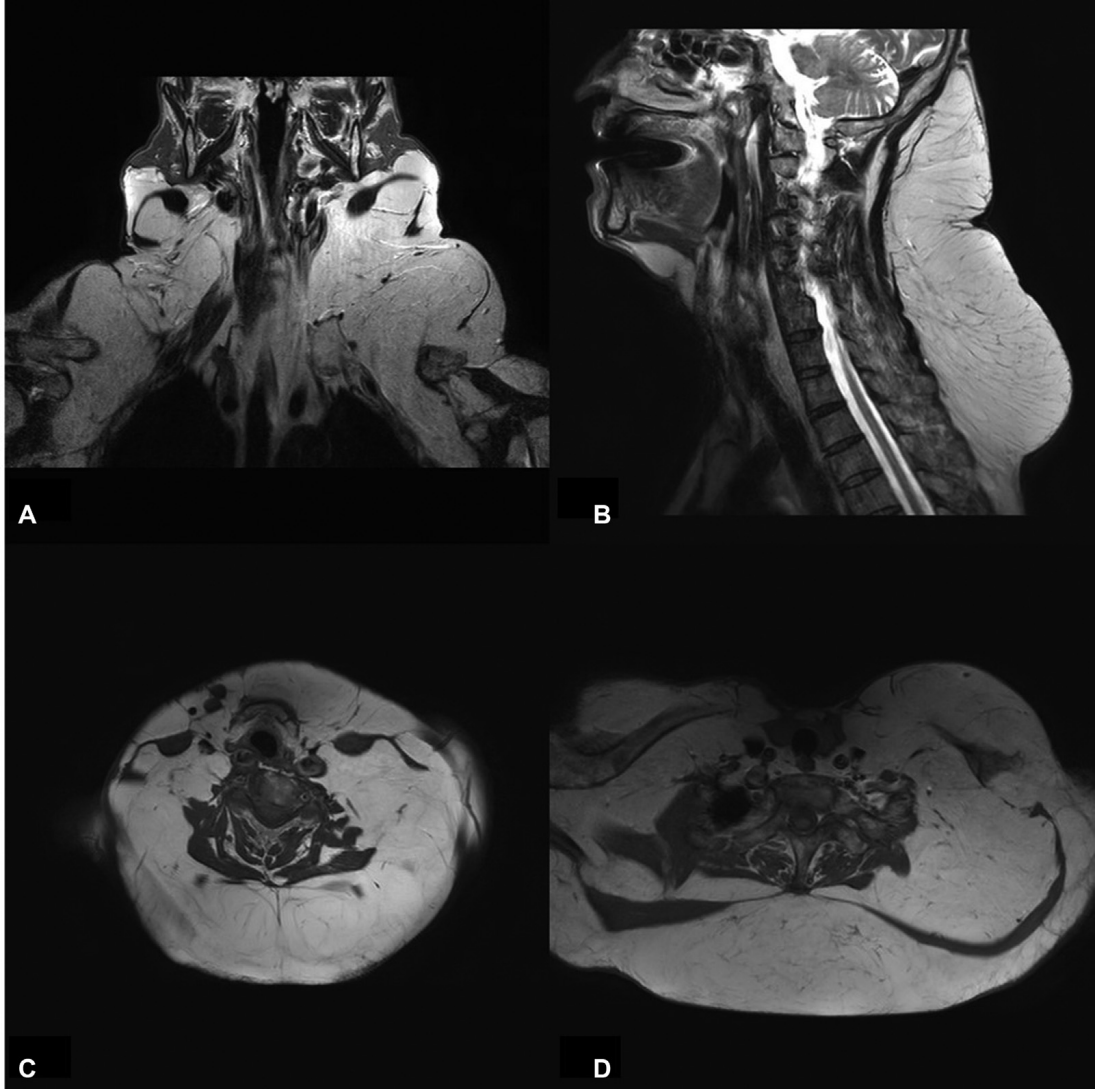
Preoperative magnetic resonance imaging (MRI) images. Preoperative MRI revealed hypertrophic fat tissue around the neck and anterior chest wall, which was consistent with Madelung's disease ( **A** , coronal view; **B** , midsagittal view; **C, D** , axial view).

Physical examination revealed diffuse enlargement around the neck and a palpable hump on the upper back, left supraclavicular region, bilateral breasts, lower abdomen, and scrotal region. Preoperative magnetic resonance imaging (MRI) revealed significant hypertrophic fat tissue around the neck and anterior chest wall, consistent with the diagnosis of MD ( Fig. 2 ). The preoperative MRI did not reveal any significant involvement of other major structures around the cervical area and mediastinum. Therefore, we were able to focus on addressing the remarkably hypertrophic fat tissue at the neck and chest wall. The MRI provided valuable information on the location and extent of these abnormalities, which guided our decision-making process regarding the appropriate surgical approach and the extent of resection needed. Specifically, we utilized imaging to plan our incisions and to identify the best trajectory for accessing the affected tissues while minimizing damage to surrounding structures.

**Fig. 3:**
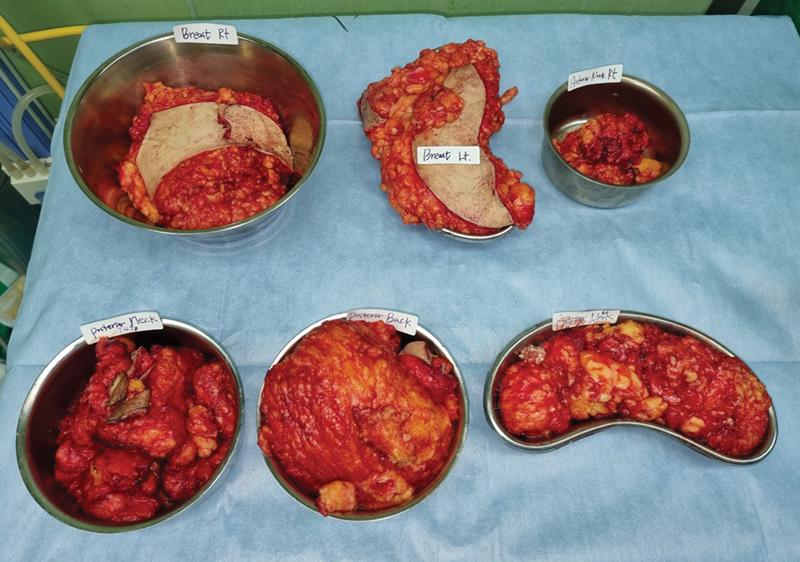
Intraoperative image of specimens. Large fatty masses were excised (right breast, 1,086 g; left breast 1,164 g; right anterior neck, 186 g; posterior neck, 763 g; upper back, 1,072 g; left anterior neck, 635 g).

Surgical management was planned to address the bulging neck contour and the palpable mass. Multiple linear incisions were made on the neck, and approximately 763, 186, and 635 g of posterior, right, and left cervical fat tissue were excised, respectively. A single wide, transverse incision was made on the upper back, and 1,072 g of the mass was excised. Masses in the bilateral breasts (right: 1,086 g; left: 1,164 g) were excised, preserving the inferior pedicle ( Fig. 3 ). No connective tissue capsules surrounding the lesion were observed. The removed adipose tissue was well vascularized, which made full hemostasis difficult during surgery. The remnant skin was trimmed, and the wound was closed in layers. Hemovac drains were placed. The pathological report of the masses showed mature adipose tissue, consistent with lipomatosis. Partial necrosis of the skin flap occurred in the area where lipectomy was performed on the posterior neck 14 days after surgery, which necessitated a revision procedure. The patient's recovery was optimal, and he was discharged without complications.

**Fig. 4:**
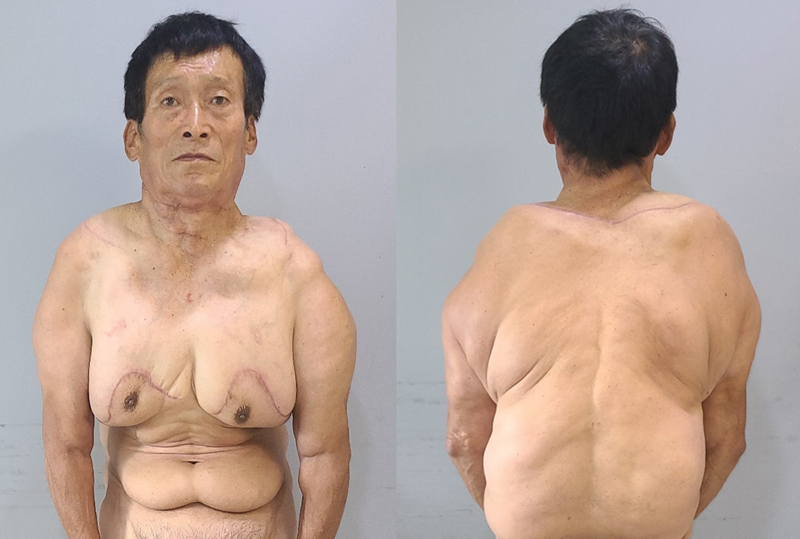
Four months' postoperation. During the 4-month postoperative follow-up period, the patient showed an improved appearance, despite the incomplete excision of excess adipose tissue.

Four months postoperatively, the patient's appearance improved, despite incomplete excision of the adipose tissue ( Fig. 4 ). We planned a second operation for the remnant mass in both upper arm and scrotum.

The patient provided written informed consent for the publication and use of images.

## Discussion

MD is a rare disorder, and only a small number of cases have been reported. Historically, MD typically affects patients in countries bordering the Mediterranean, with an estimated incidence of 1:25,000 in Italy. 5 Cases from other regions, such as Asia, have also been reported. 6 A recent systematic review of MD cases showed that majority of patients are located in Europe (79.7%), especially Portugal (37%) and Italy (21.3%). In Asian populations, only sporadic cases and a few systematic retrospective analyses have been reported in recent years. 7

Although the etiology of MD is not clearly understood, several factors have been identified in its development. These include alcoholism, HIV, 6 and other metabolic disturbances, including abnormal glucose tolerance, excessive secretion of insulin, hyperuricemia, renal tubular acidosis, alterations in liver enzyme levels, and abnormal function of the thyroid, adrenal, and pituitary gland, and testicles. 8 Although the exact pathophysiology has not been identified, it has been proposed that MD is caused by a block in catecholamine-stimulated lipolysis. 4

The diagnosis of MD may be established using clinical data, physical examination, and imaging such as computed tomography, 5 ultrasonography, or MRI. 7 9 The images show symmetrically distributed unencapsulated fatty tissue accumulating within the subcutaneous tissue along the vascular and muscular planes. Other diseases, such as morbid obesity, lipoma, liposarcoma, neurofibroma, and salivary gland disorders, should be ruled out.

MD can be classified into two types according to Enzi's classification, 8 which is the most widely accepted method based on the anatomical distribution of adipose tissue. In type 1, fatty deposits are symmetrically concentrated in the neck (Madelung's collar), shoulders, supraclavicular triangle, and proximal upper limbs. In type 2, deposits occur toward more caudal regions, such as the abdomen and thighs, without affecting the neck area, which can be confused with typical obesity. In this case, both types 1 and 2 were present.

We agree with most authors that the surgical approach is the best management. Surgical treatment includes excision and liposuction, the choice of which depends on the volume and location of the fat deposits and the surgeon's preference. Lipectomy allows for complete excision but requires accurate recognition of critical structures and hemostasis. Using this method, fatty deposits can be identified despite deep infiltration with better control of bleeding. Although liposuction techniques (classic or ultrasound-assisted) appear to be far less invasive than open surgery, they have significant limitations in terms of accessibility of deep and delicate regions. Additionally, there was a tendency for a higher relapse rate in liposuction procedures compared with lipectomy (20 vs. 14.1%). 2 A systematic review of Liu et al reported the overall MD recurrence rate was 18.3%, with 18.8% following lipectomy intervention, 19.4% for liposuction, and 7.4% for the combined therapy. During the follow-up period, the mean recurrence time was 6.3 years (7 months to 10 years) in the lipectomy group and 2.6 years (7 months to 6.4 years) in the liposuction group. 10 According to previous studies, seroma and hematoma are the most common complications after surgery. Other reported complications include infection, skin necrosis, temporary facial palsy, and numbness. 2 10 In this case, partial skin necrosis occurred in the posterior neck area, but no other complications were observed.

In general, due to the difficulty of removing a large amount of fat tissue in one operation, multistage operations are often performed. 11 In this case, the team was able to successfully remove approximately 4,900 g of fatty tissue from the neck and upper body in a one-stage operation without complications such as facial nerve or vessel injury. In addition, for aesthetics, we tried to achieve as much symmetry as possible, and the patient also showed great satisfaction.

It is necessary to investigate the etiopathogenesis of MD to develop new management plans that may reduce the recurrence rate and complication rates.
